# Electrocatalytic Degradation of Phenolic Wastewater Using a Zero-Gap Flow-Through Reactor Coupled with a 3D Ti/RuO_2_-TiO_2_@Pt Electrode

**DOI:** 10.3390/molecules29051182

**Published:** 2024-03-06

**Authors:** Yunqing Zhu, Kaiyue Wen, Bingqing Li, Yirong Hao, Jianjun Zhou

**Affiliations:** School of Environmental Science and Engineering, Shaanxi University of Science and Technology, Xi’an 710021, China

**Keywords:** zero-gap flow-through reactor, electrocatalytic oxidation, 3D porous Ti/RuO_2_-TiO_2_@Pt electrode, phenolic wastewater

## Abstract

In this study, the performance of a zero-gap flow-through reactor with three-dimensional (3D) porous Ti/RuO_2_-TiO_2_@Pt anodes was systematically investigated for the electrocatalytic oxidation of phenolic wastewater, considering phenol and 4-nitrophenol (4-NP) as the target pollutants. The optimum parameters for the electrochemical oxidation of phenol and 4-NP were examined. For phenol degradation, at an initial concentration of 50 mg/L, initial pH of 7, NaCl concentration of 10.0 g/L, current density of 10 mA/cm^2^, and retention time of 30 min, the degradation efficiency achieved was 95.05%, with an energy consumption of 15.39 kWh/kg; meanwhile, for 4-NP, the degradation efficiency was 98.42% and energy consumption was 19.21 kWh/kg (at an initial concentration of 40 mg/L, initial pH of 3, NaCl concentration of 10.0 g/L, current density of 10 mA/cm^2^, and retention time of 30 min). The electrocatalytic oxidation of phenol and 4-NP conformed to the pseudo-first-order kinetics model, and the *k* values were 0.2562 min^−1^ and 0.1736 min^−1^, respectively, which are 1.7 and 3.6-times higher than those of a conventional electrolyzer. Liquid chromatography–mass spectrometry (LC–MS) was used to verify the intermediates formed during the degradation of phenol or 4-NP and a possible degradation pathway was provided. The extremely narrow electrode distance and the flow-through configuration of the zero-gap flow-through reactor were thought to be essential for its lower energy consumption and higher mass transfer efficiency. The zero-gap flow-through reactor with a novel 3D porous Ti/RuO_2_-TiO_2_@Pt electrode is a superior alternative for the treatment of industrial wastewater.

## 1. Introduction

Phenolic wastewater is a common industrial wastewater that is mainly produced in industries such as coking, oil refineries, petrochemicals, etc. [1,2]. Due to their highly toxic and refractory properties, phenolic compounds can seriously destroy ecological systems and adversely affect human health [3,4]. Therefore, rapid and highly efficient technologies for the decomposition of phenolic compounds are urgently needed. Presently, the primary methods used include adsorption [5], biodegradation [6], Fenton oxidation [7], catalytic ozonation [8], electrocatalytic oxidation [9,10], and so on. Among them, electrocatalytic oxidation is widely used for the treatment of refractory wastewater due to the advantages of its high efficiency, simple operation, environmental friendliness, and small footprint [11].

Numerous types of mixed metal oxide (MMO) anodes have been employed in the electrochemical oxidation process for the removal of phenolic pollutants [12]. In particular, RuO_2_-based anodes with excellent catalytic activity and stability are the most attractive electrocatalysts. Barışçı et al. compared the electrocatalytic oxidation of phenol with various MMO anodes [13]; among them, the Ti/RuO_2_ anode and the Ti/IrO_2_-RuO_2_ anode obtained the highest degradation efficiencies of 87.1% and 84.7%, respectively. Can O. T. et al. investigated MMO electrodes for the electrochemical oxidation of Bisphenol A (BPA) [14]; they observed that at a low current density of 25 mA/cm^2^, the Total Organic Carbon (TOC) removal efficiencies of the MMO electrodes were 61% (Pt), 54% (RuO_2_-IrO_2_), 53% (RuO_2_-TiO_2_), 50% (IrO_2_-Ta_2_O_5_), and 49% (Pt-IrO_2_), respectively. Despite the widespread employment of RuO_2_-based anodes in the electrochemical oxidation of phenol pollutants, anode surfaces appear to have cracks, which is a typical feature of the thermal deposition method [15]. These cracks can cause the metal oxide layer to be corroded by the electrolyte, resulting in the deactivation of the anode. Based on this, we combined modified microemulsion technology with a calcination process to prepare a novel encapsulated Ti/RuO_2_-TiO_2_@Pt anode that could effectively resist electrolyte corrosion and improve electrocatalytic activity and stability [16]. Meanwhile, we attempted to use a porous titanium substrate in order to increase catalyst loading and further enhance the binding between the substrate and the active layer [17]. In addition, the 3D porous Ti/RuO_2_-TiO_2_@Pt electrode has a larger electrochemical active area, which provides more active sites and greatly improves the efficiency of electrocatalytic oxidation [18].

As well as having highly efficient electrodes, the structure of electrochemical reactors is another key factor in achieving highly efficient electrocatalytic oxidation technologies and has been attracting more attention in recent years. Conventional electrolyzers are mainly used as electrocatalytic oxidation reactors and can be easily scaled up for industrial water treatment. However, the disadvantages of their low mass transfer efficiency and high energy consumption hinder their widespread application. A feasible approach to solving this problem is narrowing the inter-electrode gap (IEG) [19]. Alrehaili et al. reported the use of a microfluidic flow-by reactor with an extremely narrow IEG (about 40 µm) that showed a decrease in energy consumption by an order of magnitude compared with a conventional electrolyzer, even in the absence of supporting electrolytes [20]. Beyond this, Gonzaga et al. found that in a narrow IEG reactor, when fluid flows through the porous electrodes, the reaction performance can be greatly enhanced, and a higher reaction rate (2–4 times) and TOC degradation efficiency can be achieved [21]. This excellent performance was attributed to both improved mass transport (flow-through mobility pattern) and decreased energy consumption (narrower IEG) [22]. The flow-through reactor is a promising approach for the development of electrochemical technologies due to its high mass transfer and low energy consumption. However, for the practical application of zero-gap flow-through reactors, the production of 3D porous anodes with excellent stability and low manufacturing costs is still an insurmountable challenge.

In this study, a zero-gap flow-through reactor with a 3D porous Ti/RuO_2_-TiO_2_@Pt electrode was constructed and evaluated by degrading phenolic wastewater. In this reactor, phenol and 4-NP were used as the target pollutants, and the effects of factors including the electrolyte type and concentration, current density, initial concentration of pollutants, and pH on the degradation efficiency of the pollutants were investigated. Under optimal conditions, the reaction rate and energy consumption of the zero-gap flow-through reactor were compared with those of a conventional electrolyzer for phenol and 4-NP degradation. Finally, the intermediates of phenol and 4-NP in the electrochemical oxidation process were analyzed by LC–MS, and possible degradation pathways were proposed.

## 2. Results and Discussion

### 2.1. Effect of Electrolyte Type and Concentration

**Figure 1:**
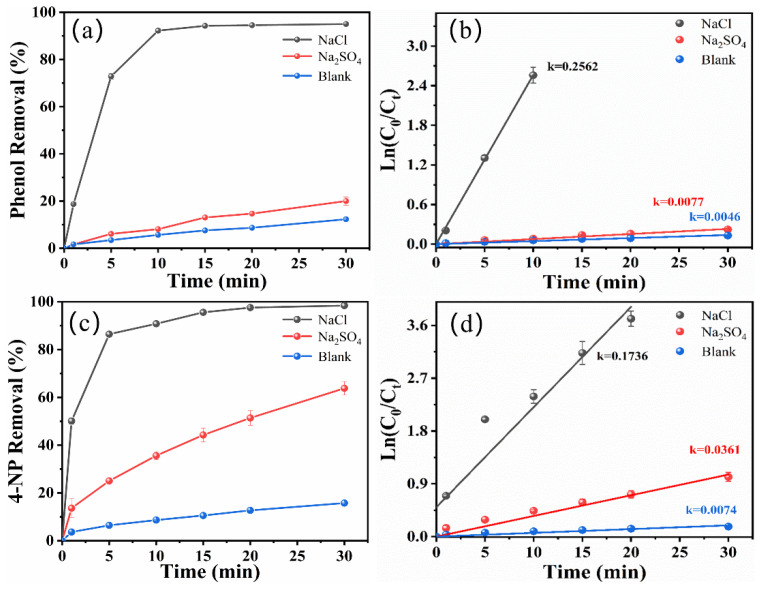
Degradation of phenol (**a**) and 4-NP (**c**) with NaCl, Na_2_SO_4,_ and the blank solution as electrolytes. Pseudo-first-order kinetic linear fitting of phenol (**b**) and 4-NP (**d**) degradation (initial concentration of target pollutant: 50 mg/L for phenol and 40 mg/L for 4-NP; current density: 10 mA/cm^2^; initial pH: 7 for phenol and 3 for 4-NP).

Figure 1 shows the degradation of phenol and 4-NP in a zero-gap flow-through reactor with either NaCl, Na_2_SO_4_, or a blank solution as the electrolyte. When NaCl was used as the electrolyte, the degradation efficiency of the phenol and 4-NP reached 95.05 ± 0.032% and 98.42 ± 0.44%, respectively (Figure 1a,c). In contrast, the degradation of phenol and 4-NP was merely 19.99% and 63.81% when using Na_2_SO_4_ as the electrolyte. The degradation efficiency of phenol and 4-NP were only 12.30 ± 0.42% and 15.75 ± 0.36% in the blank solution (without NaCl or Na_2_SO_4_ electrolytes). As shown in Figure 1b,d, the degradation of phenol and 4-NP in different electrolytes conformed to the pseudo-first-order kinetics model. As seen in Tables S1 and S2, the *k* values for the degradation of phenol and 4-NP were higher in NaCl solution (0.2562 min^−1^ and 0.1736 min^−1^, respectively). While the *k* value was lowest in the blank solution, this was due to the low conductivity of the solution without electrolytes, affecting the charge transfer rate and leading to a low-level reaction process. It is obvious that using NaCl as the electrolyte provides a higher degradation rate for phenol and 4-NP; this is probably attributed to the rapid oxidation of Cl^−^ at the electrode surface, which produces highly reactive chlorine and oxychlorine species (Equations (1) and (2)) and could significantly increase the degradation rate of organic matter [23,24]. Moreover, owing to the relatively high concentration of Cl^−^ in actual industrial wastewater, NaCl as an electrolyte also decreases the treatment costs.

(1) $$2{Cl}^{-}\to {Cl}_{2}+{2e}^{-}$$

(2) $${Cl}_{2}+H_{2}O\to HClO+H^{+}+{Cl}^{-}$$

**Figure 2:**
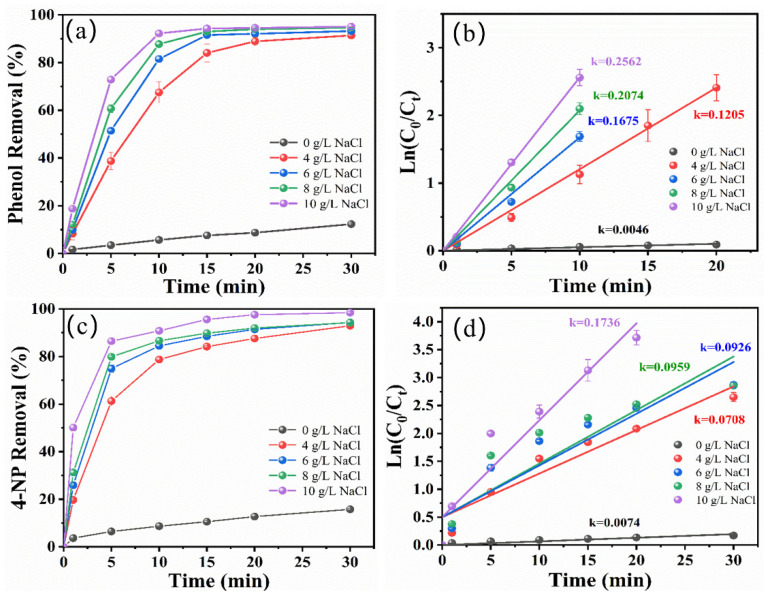
Effect of NaCl concentration on the degradation of phenol (**a**) and 4-NP (**c**). Pseudo-first-order kinetic linear fitting of phenol (**b**) and 4-NP (**d**) degradation (initial concentration of target pollutant: 50 mg/L for phenol and 40 mg/L for 4-NP, current density: 10 mA/cm^2^, initial pH: 7 for phenol and 3 for 4-NP, electrolyte: NaCl).

Subsequently, the effect of NaCl concentration (4, 6, 8, and 10 g/L) on the degradation efficiency of phenol and 4-NP was investigated in the zero-gap flow-through reactor. As shown in Figure 2a,c, the degradation rate of phenol and 4-NP increased continuously with increases in the NaCl electrolyte concentration. After 30 min of electrolysis, the degradation rate of phenol and 4-NP increased to 95.05 ± 0.035% and 98.42 ± 0.41%, respectively, when the NaCl concentration rose from 4 to 10 g/L. As shown in Figure 2b,d, the degradation of pollutants at different electrolyte concentrations conformed to the pseudo-first-order kinetics model, and the *k* value rose continuously; when the NaCl concentration was 10 g/L, the *k* values of phenol and 4-NP attained the maximum values (0.2562 min^−1^ and 0.1736 min^−1^, respectively). This result was attributed to the fact that, as the concentration of NaCl increased, the resistance of the solution decreased and the electron transfer rate accelerated, which resulted in higher *k* values and greater degradation efficiency. Meanwhile, the higher Cl^−^ concentrations also promoted higher activated chlorine production, allowing for the faster degradation rate of organic substances [25].

### 2.2. Effect of Current Density

**Figure 3:**
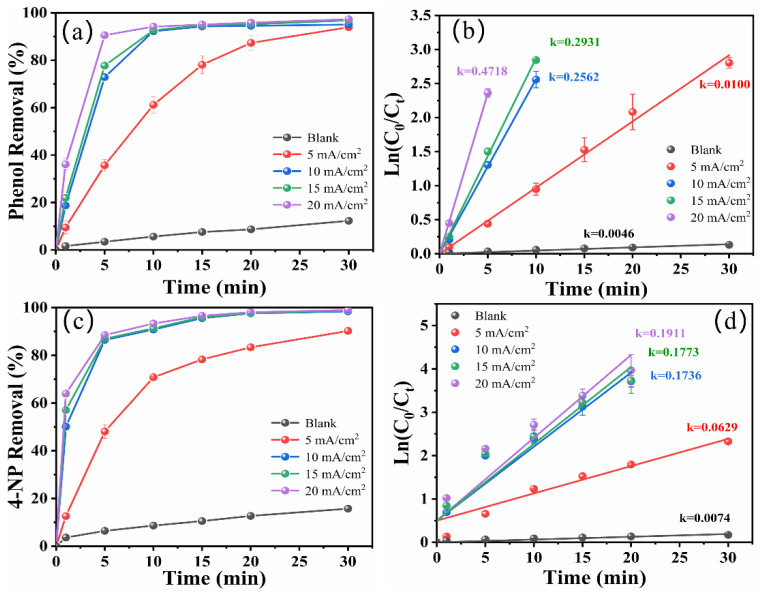
Effect of current density on the degradation of phenol (**a**) and 4-NP (**c**). Pseudo first-order kinetic linear fitting of phenol (**b**) and 4-NP (**d**) degradation (initial concentration of target pollutant: 50 mg/L for phenol and 40 mg/L for 4-NP, initial pH: 7 for phenol and 3 for 4-NP, NaCl concentration: 10 g/L).

The current density can affect the production of ·OH on the electrode surface, which consequently affects the pollutant degradation efficiency; thus, the influence of current density on the electrocatalytic reaction was studied [26]. Figure 3a shows the effect of different current densities on the degradation of phenol at an initial concentration of 50 mg/L in a zero-gap flow-through reactor. It can be seen that the percentage of phenol degradation increased from 93.94 ± 0.47% to 95.05 ± 0.081% when the current density was increased from 5 to 10 mA/cm^2^. However, when the current density was continuously increased to 20 mA/cm^2^, the percentage of phenol degradation only increased by 2%. As shown in Figure 3b, the degradation process of phenol conforms to the pseudo-first-order kinetics model. As seen in Table S1, for the current density of 5, 10, 15, and 20 mA/cm^2^, the corresponding *k* values were 0.0100 min^−1^, 0.2562 min^−1^, 0.2931 min^−1^, and 0.4718 min^−1^, respectively. These results can probably be ascribed to the increased generation of ·OH with increased current density at the same reaction time, which resulted in higher phenol degradation [27]. However, when current density exceeded 10 mA/cm^2^, the phenol degradation increased slowly, which may be attributed to the rapid generation of reactive species under a high current density, which accelerated the oxidation rate of the phenol. The phenol degradation process is mainly controlled by mass transportation, so there is no significant enhancement to the degradation efficiency by continuing to increase the current density [28]. In addition, the excessive current density enhanced side reactions and increased energy consumption [29]. For 4-NP degradation, as shown in Figure 3c,d, when the current density was 5, 10, 15, and 20 mA/cm^2^, the degradation percentage was 90.24 ± 0.48%, 98.42 ± 0.44%, 98.75 ± 0.53%, and 98.96 ± 0.08%, respectively. The degradation of 4-NP also conformed to the pseudo-first-order kinetics model. With increasing current density (from 5 to 20 mA/cm^2^), the *k* values were 0.0629 min^−1^, 0.1736 min^−1^, 0.1773 min^−1^, and 0.1911 min^−1^, respectively (Table S2). It can be seen that when the current density continued to increase from 10 to 20 mA/cm^2^, the degradation efficiency of 4-NP changed only slightly and the *k* value grew slowly. These results indicate that a continued increase in current density has no impact on the degradation efficiency of 4-NP. Instead, the higher current density aggravates the production of the oxygen evolution reaction [30].

### 2.3. Effect of Initial Concentration of Phenol and 4-NP

**Figure 4:**
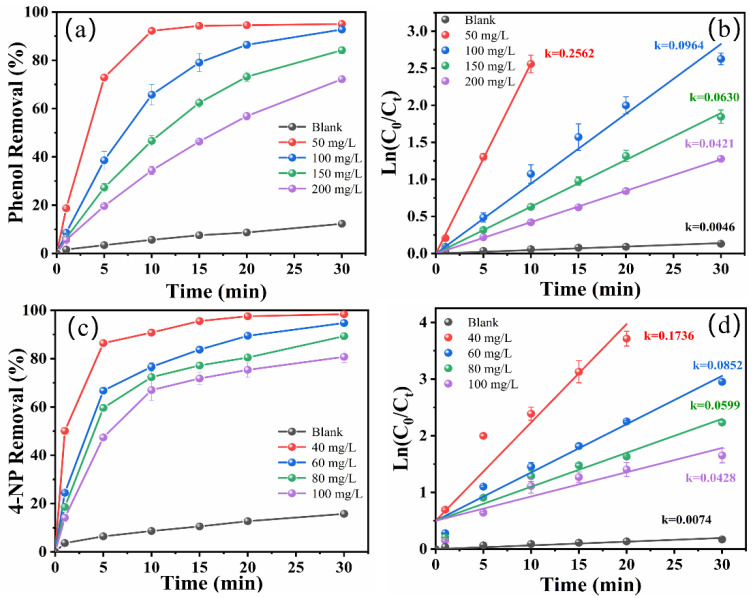
Effect of initial concentration on the degradation of phenol (**a**) and 4-NP (**c**). Pseudo first-order kinetic linear fitting of phenol (**b**) and 4-NP (**d**) degradation (current density: 10 mA/cm^2^, initial pH: 7 for phenol and 3 for 4-NP, NaCl concentration: 10 g/L).

Considering a practical application of the zero-gap flow-through reactor for the treatment of phenolic wastewater, the effect of different initial concentrations of phenol or 4-NP on the degradation efficiency was investigated. As shown in Figure 4a,c, when the phenol concentration was increased from 50 to 200 mg/L, the percentage of phenol degradation decreased from 95.05 ± 0.035% to 72.14 ± 0.047% within a 30 min reaction. When the concentration of 4-NP was increased from 40 to 100 mg/L, the degradation percentage of 4-NP decreased from 98.42 ± 0.44% to 80.77 ± 1.47% within a 30 min reaction. This indicates that the higher the initial concentration of the pollutant, the more time it takes to degrade it. Based on Figure 4b,d, the degradation of phenol and 4-NP at various concentrations conformed to the pseudo-first-order kinetic model, and the *k* value gradually decreased with increases in the initial concentration (Tables S1 and S2). The non-homogeneous reaction was a multi-step and complicated process involving chemical reaction kinetics and diffusion kinetics. The initial phenol concentration increase aided the diffusion of phenol to the electrode surface, but the oversaturation of adsorbed phenol led to a reduction in phenol involved in the reaction, resulting in a significant decrease in the *k* value [31]. In addition, excessively adsorbed phenol also causes a reduction in the generation of ·OH radicals on the electrode surface, decreasing the degradation efficiency.

### 2.4. Effect of Initial pH

**Figure 5:**
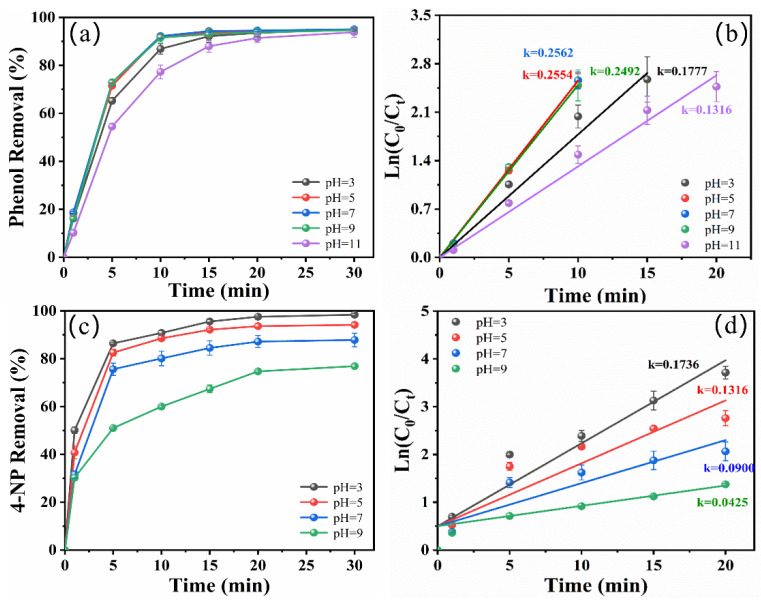
Effect of initial pH on the degradation of phenol (**a**) and 4-NP (**c**). Pseudo first-order kinetic linear fitting of phenol (**b**) and 4-NP (**d**) degradation (initial concentration of target pollutant: 50 mg/L for phenol and 40 mg/L for 4-NP, current density: 10 mA/cm^2^, NaCl concentration: 10 g/L).

The initial pH is an important factor for electrochemical oxidation that can affect the performance of electrochemical degradation. The effect of different initial pHs on phenol and 4-NP degradation efficiency was investigated in the zero-gap flow-through reactor. As shown in Figure 5a,b, there was little difference between the degradation percentages of phenol at varying initial pH values, with almost all achieving about 95% degradation in a 30 min reaction and all reactions following pseudo-first-order kinetics. When the initial pH was 7, the phenol degradation percentage reached its maximum and the *k* value was 0.2562 min^−1^ (Table S1). When the pH was reduced to 3, the *k* value decreased to 0.1777 min^−1^. These findings are attributed to the fact that active chlorine mainly exists as Cl2 at lower pH values and its oxidation capacity is weaker than that of ClO^−^ [32]. When the pH was raised to 11, the *k* value was only 0.1316 min^−1^. An alkaline environment leads to decreased oxygen evolution potential and intensifies the occurrence of side reactions of oxygen evolution [33]. Therefore, pH 7 was well suited for the electrocatalytic degradation of phenol. As shown in Figure 5c,d, when the initial pH of the solution was increased from 3 to 9, the degradation efficiency of 4-NP decreased from 98.42 ± 0.44% to 76.92 ± 0.81%, and the *k* value decreased from 0.1736 min^−1^ to 0.0425 min^−1^ (Table S2). The results illustrated that 4-NP was more favorably degraded in acidic solutions. Consistent with previous studies, increasing pH values resulted in decreased oxygen evolution potential, and some of the ·OH combined with OH^−^ to form water as the OH^−^ concentration increased during alkaline conditions [9]. Therefore, less ·OH was used to degrade the 4-NP contaminants.

### 2.5. Reactor Comparison

**Figure 6:**
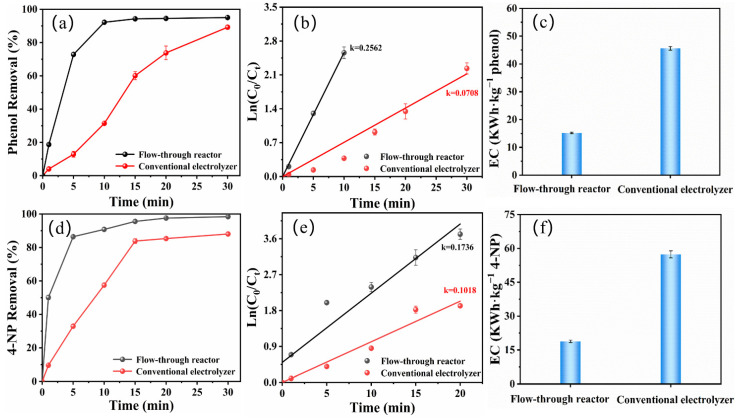
Degradation of phenol and 4-NP in zero-gap flow-through reactor and conventional electrolyzer under the same conditions. degradation of phenol (**a**) and 4-NP (**d**). Pseudo first-order kinetic linear fitting of phenol (**b**) and 4-NP (**e**) degradation. Electrical energy consumption required after 90% degradation of phenol (**c**) and 4-NP (**f**) in both reactors.

Figure 6 compares the degradation of phenol and 4-NP in a zero-gap flow-through reactor and a conventional electrolyzer under optimal conditions. In the zero-gap flow-through reactor, the degradation percentage of phenol and 4-NP was above 95% at a current density of 10 mA/cm^2^, while in a conventional electrolyzer, the degradation percentage was approximately 88% in a 30 min reaction (Figure 6a,d). Figure 6b,e show the kinetic decay of phenol and 4-NP, which in all cases, adapted well to the pseudo-first-order kinetic model. The *k* values were 0.2562 min^−1^ and 0.1736 min^−1^ for the degradation of phenol and 4-NP in the zero-gap flow-through reactor, respectively (Tables S1 and S2). In contrast, the *k* values were only 0.0708 min^−1^ and 0.1018 min^−1^ for the degradation of phenol and 4-NP in the conventional electrolyzer. When using the zero-gap flow-through reactor, the degradation rate was much faster (1.7–3.6 times) than that in the conventional electrolyzer. The zero-gap flow-through reactor promoted significantly faster and higher degradation of the target pollutants. Consistent with previous studies, this fact can be explained by the structure of the zero-gap flow-through reactor and the use of 3D electrodes [34,35,36]. The narrower IEG configuration in a zero-gap flow-through reactor decreases the ohmic resistance of the system and also improves the mass transport efficiency. Generally, the extremely narrow distance between electrodes automatically enhances the mass transport of contaminants to the electrode surface [21]. In addition, the fluid percolates through the 3D electrodes, providing a larger contact area between the phenol and 4-NP molecules and the electrodes, which promotes the transfer of phenolic compounds to its surface and, therefore, improves the degradation rate of phenol and 4-NP [22,37,38].

To compare two reactors in terms of the energy consumption required to remove a unit mass of pollutant, it was necessary to use eq 5 to calculate the energy consumption required for the degradation of phenol and 4-NP in the two reactors. Figure 6c,f show the energy consumption for the degradation of approximately 90% of the phenolic compounds in the conventional electrolyzer and the zero-gap flow-through reactor, respectively. With the same electrolysis conditions, the zero-gap flow-through reactor reduces energy consumption by approximately 66% compared to conventional electrolyzers for the degradation of phenol and 4-NP. The high energy consumption of the conventional electrolyzer compared to the zero-gap flow-through reactor (0.1 mm) is associated with high ohmic losses due to the larger distance (about 20 mm) between the anode and cathode. This conclusion agrees with previous reports saying that the performance of the flow-through reactor is superior to that of a conventional electrolyzer in terms of its lower ohmic losses [21,22,37]. This indicates that the 3D porous electrodes configured into the zero-gap flow-through reactor not only resulted in the increased degradation efficiency of phenolic contaminants, but also decreased energy consumption.

### 2.6. Degradation Pathways of Phenol and 4-NP

LC–MS was used to analyze the water samples after different degradation times to detect the intermediate products present during the degradation of phenol and 4-NP and to determine their degradation pathways. Figures S1 and S2 show the mass spectra of the intermediate products produced during the degradation of phenol and 4-NP, which are consistent with those reported previously. Based on the detected intermediates, the degradation process (Figure S3) and possible reaction pathways for the electrochemical oxidation of phenol and 4-NP are proposed [9,39,40].

**Figure 7:**
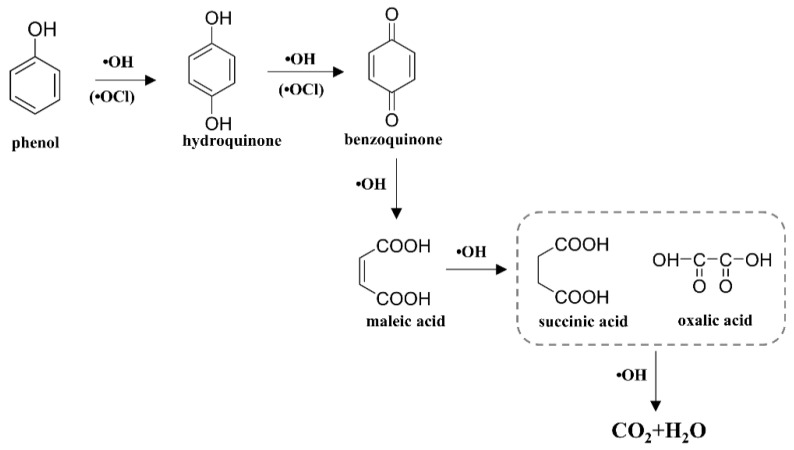
Proposed degradation pathway of phenol during electrochemical degradation in a zero-gap flow-through reactor.

The electrocatalytic oxidation degradation of phenolic pollutants occurs primarily through radicals with strong oxidative capabilities, produced by the anode in the reaction process to oxidize and decompose the pollutants [41]. In the electrocatalytic oxidation process, phenol can be easily reacted with reactive groups such as ·ClO and ·OH to form phenol hydroxylation products and other byproducts due to the properties of its electrophilic groups [42]. For the monocyclic structure of phenol, the ·OH radical is more likely than reactive chlorine to react with the benzene ring to form C_6_H_6_O_2_ (*m*/*z* = 111.04). Subsequently, the ·OH radical continues to attack the ·OH functional group of C_6_H_6_O_2_ and deprives it of hydrogen atoms to form C_6_H_4_O_2_ (*m*/*z* = 109.02), which is further oxidized, and then the ring cleavage reaction occurs. Thereafter, the C_4_H_4_O_4_ (*m*/*z* = 117.01) produced through ring cleavage is further converted to smaller organic acids such as C_2_H_2_O_4_ and C_4_H_6_O_4_ (*m*/*z* = 119.03). Finally, the organic acids are oxidized to CO_2_ and H_2_O. The potential degradation pathways of phenol were proposed based on the above analyses and are shown in Figure 7.

**Figure 8:**
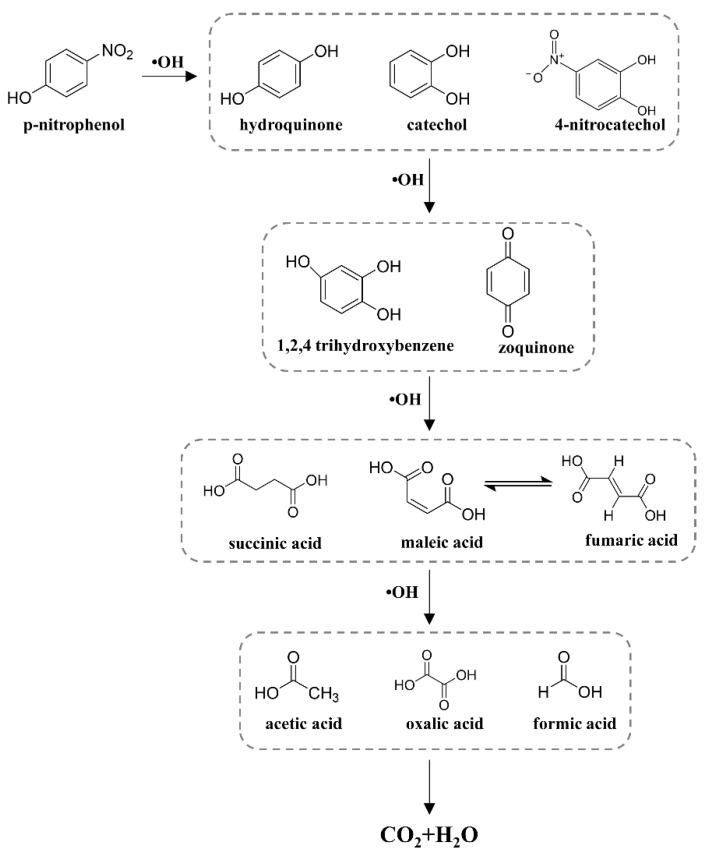
Proposed degradation pathway of 4-NP during electrochemical degradation in zero-gap flow-through reactor.

Based on the intermediates at different time periods, the possible degradation pathway of 4-NP was proposed. As shown in Figure 8, the first step is the separation of the nitro group in 4-NP from the benzene ring, resulting in the formation of aromatic derivatives including C_6_H_6_O_2_ (*m*/*z* = 111.04) and C_6_H_5_NO_4_ (*m*/*z* = 156.02). The aromatic derivatives are attacked by ·OH to form C_6_H_6_O_3_ (*m*/*z* = 127.03) and C_6_H_4_O_2_ (*m*/*z* = 109.02) and are subsequently degraded to organic acids such as C_4_H_4_O_4_ (*m*/*z* =117.01) and C_4_H_6_O_4_ (*m*/*z* = 119.03) via a ring cleavage reaction. These organic acids are further oxidized to smaller molecular acids and finally oxidized to CO_2_ and H_2_O.

## 3. Experiment

### 3.1. Chemical Reagents and Materials

Ruthenium chloride trihydrate (RuCl_3_·3H_2_O) and chloroplatinic acid (H_2_PtCl_6_·6H_2_O) were purchased from Guiyang Platinum Industry Co., Ltd. (Guiyang, China). Titanium trichloride (TiCl_3_) and ethanol (C_2_H_5_OH) were purchased from Aladdin Reagent Inc. (Shanghai, China). Cetyltrimethylammonium bromide (CTAB), toluene (C_7_H_8_), isopropanol (C_3_H_8_O), citric acid (C_6_H_8_O_7_), sodium hydroxide (NaOH), and acetone were supplied by Sinopharm (Beijing, China). The porous titanium plates with 99.7% purity and 0.5 mm thickness were obtained from Baoji Titanium Industry Co., Ltd. (Baoji, China). All chemical reagents were of analytical grade and used without further purification.

### 3.2. Preparation of Electrodes

The 3D porous Ti/RuO_2_-TiO_2_@Pt electrode (4.6 cm × 4.6 cm) was prepared using the method described in our previous work [17]. Firstly, Pt/Ru-Ti composite oxide nanoparticles prepared via the microemulsion method were added to isopropanol to form a uniformly dispersed coating solution. The pre-treated porous titanium substrate was dipped in the coating solution and calcined in a muffle furnace at 350 ℃ for 15 min, and then the above steps were repeated several times. Finally, the electrodes were annealed in a muffle furnace at 450 ℃ for 2 h to achieve a 3D porous Ti/RuO_2_-TiO_2_@Pt electrode. The detailed structural characteristics and the lifetime tests of the 3D porous Ti/RuO_2_-TiO_2_@Pt electrode are presented in the Supporting Information (Figures S4 and S5). 

### 3.3. Zero-Gap Flow-Through Reactor

Figure S6a shows a diagram of the zero-gap flow-through reactor with a size of 7.4 × 3.0 × 7.4 cm. The as-prepared 3D porous Ti/RuO_2_-TiO_2_@Pt electrode was used as the anode, while a porous titanium plate was adopted as the cathode, and the anode and cathode were separated by a macroporous separator with a thickness of 0.1 mm. The wastewater was pumped into the reactor by a peristaltic pump and fed perpendicular to the electrodes, flowing through the anode and cathode. The total volume of simulated wastewater for recirculation through the reactor was 200 mL, and the pump flow rate was 17.5 L/h, which corresponds to a hydraulic retention time (HRT) of 2.66 s. The reactor was connected to a DC-regulated power supply (DH1718E-6, Shanghai Chenhua Instruments Co., Ltd., Shanghai, China) and the current density was controlled by varying the output current of the DC power supply. The samples were collected at the outlet with a fixed time interval.

The phenol and 4-NP degradation performances were compared for the different reactors (zero-gap flow-through and conventional electrolyzer) with identical electrodes under similar reaction conditions. Figure S6b shows the diagram of the conventional electrolyzer. The experiments were conducted in a tank containing 200 mL of simulated wastewater with magnetic stirring at 700 rpm to promote target contaminant transport towards the electrode surface. The interelectrode distance was 2 cm, and the reaction times were 30 min for all reactions.

### 3.4. Analytical Methods

The variation in the phenol concentration was detected by ultra high-performance liquid chromatography (UPLC, Hclass, Water, Milford, MA, USA) with a BET C18 column (2.1 × 100 mm, 1.7 μm). A mixture of 60% methanol and 50% acidic pure water was used as a mobile phase, flowing at 0.1 mL/min. The injection volume was 10.0 μL, and the detection wavelength of the UV detector was 270 nm. For the determination of 4-NP concentration, the mobile phase consisted of methanol/water (*v*/*v*) at 70/30 and the UV detector wavelength was set at 280 nm. 

The intermediates in the degradation process were determined using liquid chromatography–mass spectroscopy coupled with a triple-stage quadrupole mass spectrometry (LC–MS, Q-extraction method, Thermo, Waltham, MA, USA) equipped with a Hypersil GOLD column (2.1 × 100 mm, 1.9 µm). A 2 μL sample was injected using the autosampler. The mobile phase was initially 0.2% formic acid and acetonitrile at a flow rate of 0.3 mL/min. The MS analysis was adjusted to positive electrospray ionization (ESI(+)) mode, and the relevant MS data were collected by scanning between *m*/*z* 100 and 500.

The phenol or 4-NP degradation efficiency and its kinetics were calculated using Equations (3) and (4):

(3) $$\eta \left(\%\right)=\frac{\left(C_{0}-C_{t}\right)}{C_{0}}\times 100\%$$

(4) $$Ln(C_{0}/C_{t})=kt$$

where *η* is the degradation efficiency (%); C_0_ and C_t_ (mg·L^−1^) represent the initial concentration and the concentration at a certain time after phenol or 4-NP degradation, respectively; and *k* (min^−1^) represents the pseudo-first-order kinetic coefficient.

To compare the conventional electrolyzer with the zero-gap flow-through reactor, the energy consumption was estimated for the degradation of pollutants. The energy consumption (EC) is denoted by kWh/kg, which is the amount of electrical energy consumed per 1 kg of pollutant removed, and is calculated using Equation (5):

(5) $$EC=1000\times \frac{UIt}{V\times \left(C_{0}-C_{t}\right)}$$

where U is the average potential (V); I is the applied current (A); *t* is the reaction time (h); and V is the solution volume treated (m^3^).

## 4. Conclusions

In this work, a zero-gap flow-through reactor was constructed with a 3D porous Ti/RuO_2_-TiO_2_@Pt electrode and used for the electrocatalytic oxidation of phenolic wastewater. At a current density of 10 mA/cm^2^, the zero-gap flow-through reactor could achieve an about 90% degradation of phenol and 4-NP in 10 min, indicating that the use of the zero-gap flow-through reactor offers a fast degradation rate without requiring high currents. The zero-gap flow-through reactor equipped with 3D anodes was proven to have a faster reaction rate and require less energy in the treatment of phenolic wastewater because of the narrow inter-electrode gap (reduced ohmic resistance of the system) and the flow-through configuration (improved mass transfer). In addition, during the electrocatalytic degradation process with NaCl, phenols were primarily oxidized by in situ-generated ·ClO and ·OH radicals, producing a series of intermediates that could be further decomposed. The design of the reactor is of great significance for improving the efficiency of the treatment processes, and reactors equipped with 3D anodes have proven to be extremely effective in removing phenols. Therefore, a zero-gap flow-through reactor with 3D porous Ti/RuO_2_-TiO_2_@Pt as an anode would have excellent prospects for the treatment of phenolic wastewater.

## Data Availability

Data are contained within the article and Supplementary Materials.

## Supplementary Materials

The following supporting information can be downloaded at: https://www.mdpi.com/article/10.3390/molecules29051182/s1, Figure S1: LC–MS spectrum for mentioned intermediates of phenol; Figure S2: LC-MS spectrum for mentioned intermediates of 4-NP; Figure S3: Schematic diagram of the electrochemical oxidative degradation process for phenolic pollutants; Figure S4: XRD patterns of porous Ti/RuO_2_-TiO_2_@Pt (a), SEM images of porous Ti/RuO_2_-TiO_2_@Pt at different magnifications (b,c), EDS elemental mappings (d) and EDS spectrum (e) of porous Ti/RuO_2_-TiO_2_@Pt, (f) Mercury compression analysis of porous Ti/RuO_2_-TiO_2_@Pt; Figure S5: The electric double-layer capacitance diagram (a), LSV curves (b), Tafel curves (c) of porous Ti/RuO_2_-TiO_2_@Pt and Ti/RuO_2_-IrO_2_ electrodes, (d) Accelerated life test of the prepared electrodes at 2 A/cm^2^ in 1 mol/L H_2_SO_4_ at 40 °C; Figure S6: Scheme of the electrochemical systems. (a) zero-gap flow-through reactor, (b) conventional electrolyzer. Anode: 3D porous Ti/RuO_2_-IrO_2_@Pt and cathode: porous titanium plate; Table S1: Parameters of the pseudo-first-order kinetic model for electrocatalytic oxidation of phenol with the zero-gap flow-through reactor; Table S2: Parameters of the pseudo-first-order kinetic model for electrocatalytic oxidation of phenol with the zero-gap flow-through reactor.
