# Supplementary material for: Electrocatalytic Degradation of Phenolic Wastewater Using a Zero-Gap Flow-Through Reactor Coupled with a 3D Ti/RuO2-TiO2@Pt Electrode
Source: Molecules. 2024 Mar 6;29(5):1182. doi: 10.3390/molecules29051182 (PMC10934669; doi:10.3390/molecules29051182)
Supplement: Supplementary file 1 [file molecules-29-01182-s001.zip › molecules-2876260-supplementary.pdf]

# Electrocatalytic Degradation of Phenolic Wastewater using a Zero-Gap Flow-through Reactor Coupled with a 3D Ti/RuO2-TiO2@Pt Electrode

**Yunqing Zhu *, Kaiyue Wen, Bingqing Li,Yirong Hao, Jianjun Zhou**

School of Environmental Science and Engineering, Shaanxi University of Science and Technology, Xian 710021, PR China

*Corresponding author: **Yunqing Zhu**

Email address: zhuyunqing@sust.edu.cn

**Table S1**

Parameters of the pseudo-first order kinetic model for electrocatalytic oxidation of phenol with the zero-gap flow-through reactor.

| pollutant | Variables | Range | k ($min^{-1}$) | $R^2$ |
|---|---|---|---|---|
| phenol | electrolyte | $Na_2SO_4$ | 0.0077 | 0.9899 |
| | | NaCl | 0.2562 | 0.9999 |
| | | Blank | 0.0046 | 0.9953 |
| | NaCl (g/L) | 4 | 0.1205 | 0.9988 |
| | | 6 | 0.1675 | 0.9981 |
| | | 8 | 0.2074 | 0.9981 |
| | | 10 | 0.2562 | 0.9999 |
| | current density ($mA/cm^2$) | 5 | 0.0100 | 0.9972 |
| | | 10 | 0.2562 | 0.9999 |
| | | 15 | 0.2931 | 0.9987 |
| | | 20 | 0.4718 | 0.9998 |
| | initial concentration (mg/L) | 50 | 0.2562 | 0.9999 |
| | | 100 | 0.0964 | 0.9921 |
| | | 150 | 0.0630 | 0.9988 |
| | | 200 | 0.0421 | 0.9997 |
| | pH | 3 | 0.1777 | 0.9930 |
| | | 5 | 0.2554 | 0.9998 |
| | | 7 | 0.2562 | 0.9999 |
| | | 9 | 0.2492 | 0.9998 |
| | | 11 | 0.1316 | 0.9942 |
| | Flow-through reactor | / | 0.2562 | 0.9999 |
| | Conventional electrolyzer | / | 0.0708 | 0.9938 |

**Table S2**

Parameters of the pseudo-first order kinetic model for electrocatalytic oxidation of 4-NP with the zero-gap flow-through reactor.

| pollutant | Variables | Range | k ($min^{-1}$ ) | $R^2$ |
|---|---|---|---|---|
| 4-NP | electrolyte | $Na_2SO_4$ | 0.0361 | 0.9806 |
| | | NaCl | 0.1736 | 0.9734 |
| | | Blank | 0.0074 | 0.9863 |
| | NaCl (g/L) | 4 | 0.0780 | 0.9664 |
| | | 6 | 0.0926 | 0.9543 |
| | | 8 | 0.0959 | 0.9386 |
| | | 10 | 0.1736 | 0.9737 |
| | current density ($mA/cm^2$) | 5 | 0.0629 | 0.9567 |
| | | 10 | 0.1736 | 0.9737 |
| | | 15 | 0.1773 | 0.9715 |
| | | 20 | 0.1911 | 0.9680 |
| | initial concentration (mg/L) | 40 | 0.1736 | 0.9737 |
| | | 60 | 0.0852 | 0.9773 |
| | | 80 | 0.0599 | 0.9587 |
| | | 100 | 0.0428 | 0.9303 |
| | pH | 3 | 0.1736 | 0.9737 |
| | | 5 | 0.1316 | 0.9525 |
| | | 7 | 0.0900 | 0.9412 |
| | | 9 | 0.0425 | 0.9263 |
| | Flow-through reactor | / | 0.1736 | 0.9737 |
| | Conventional electrolyzer | / | 0.1018 | 0.9788 |

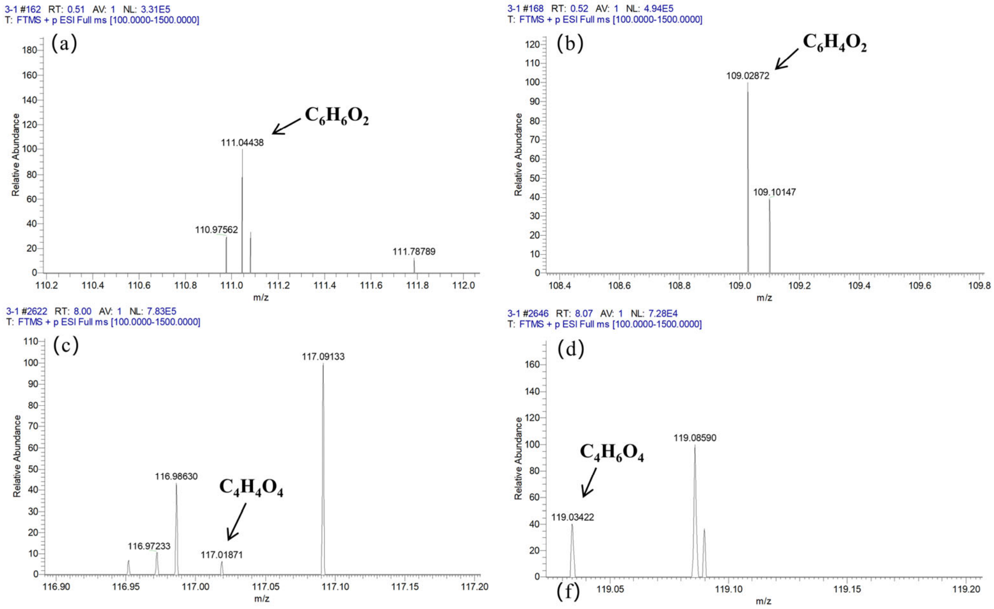


**Figure S1.** LC-MS spectrum for mentioned intermediates of phenol.

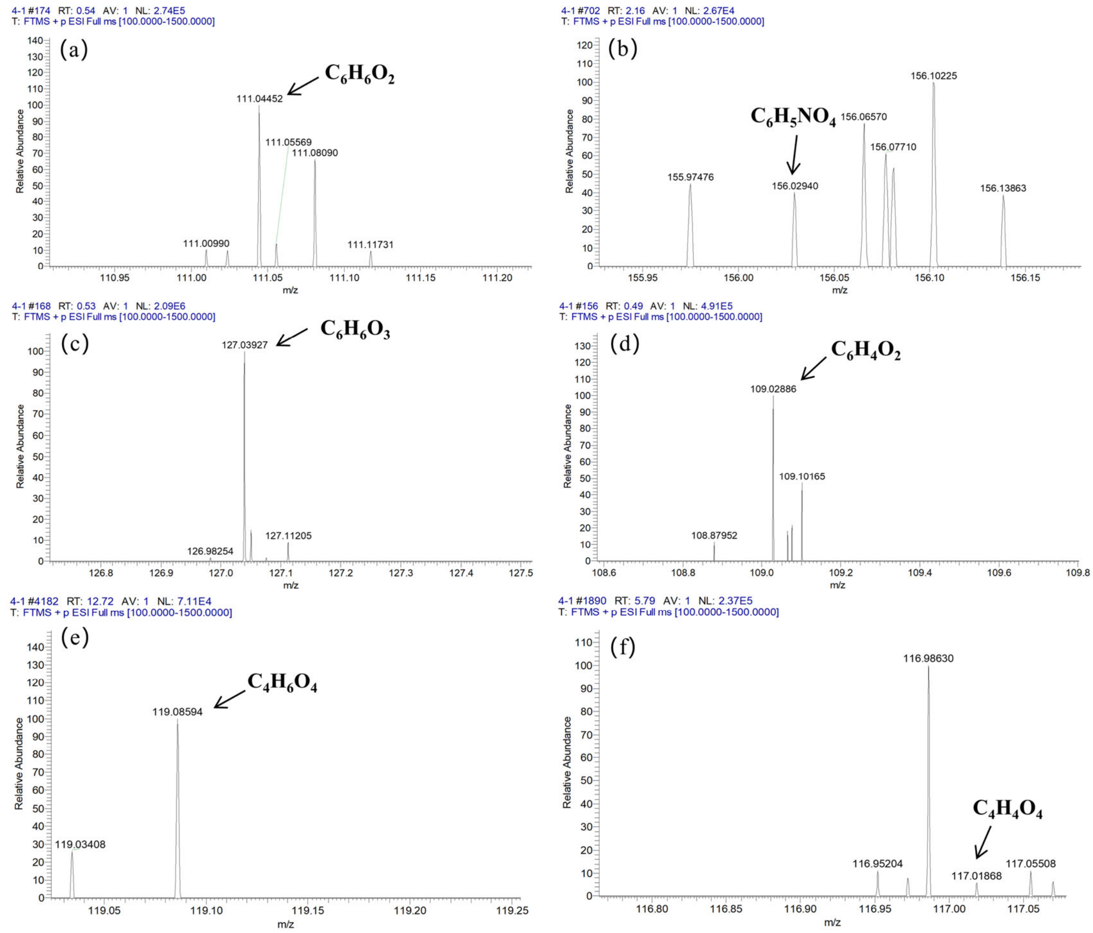


**Figure S2.** LC-MS spectrum for mentioned intermediates of 4-NP.

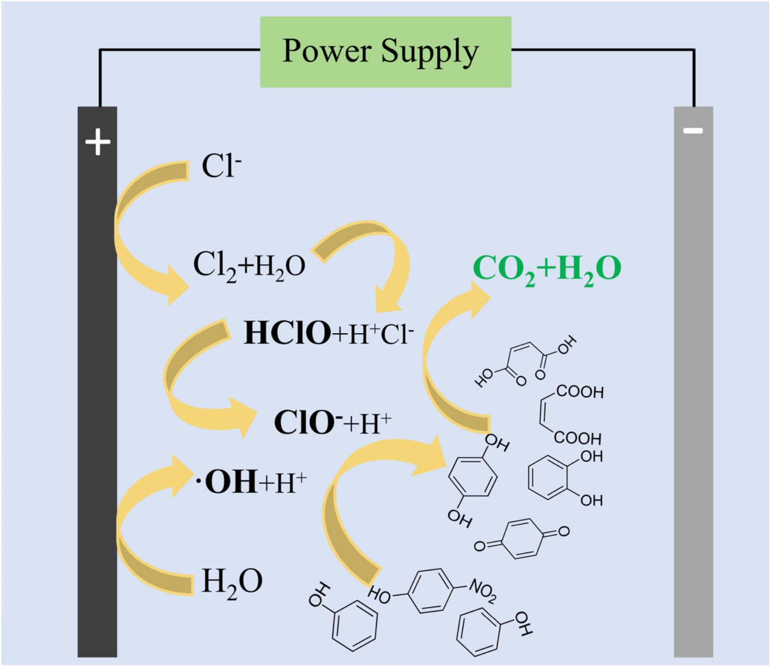


**Figure S3.** Schematic diagram of the electrochemical oxidative degradation process for phenolic pollutants.

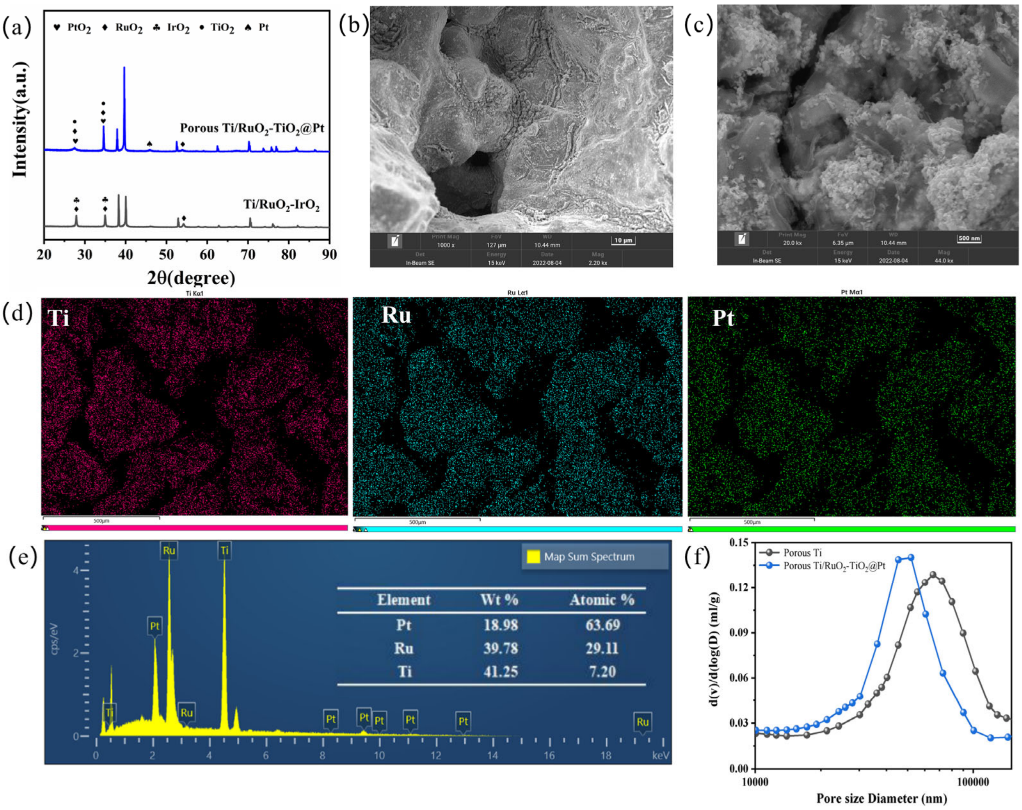


**Figure S4.** XRD patterns of porous Ti/RuO2-TiO2@Pt (a). SEM images of porous Ti/RuO2-TiO2@Pt at different magnifications (b,c). EDS elemental mappings (d) and EDS spectrum (e) of porous Ti/RuO2-TiO2@Pt. (f) Mercury compression analysis of porous Ti/RuO2-TiO2@Pt.

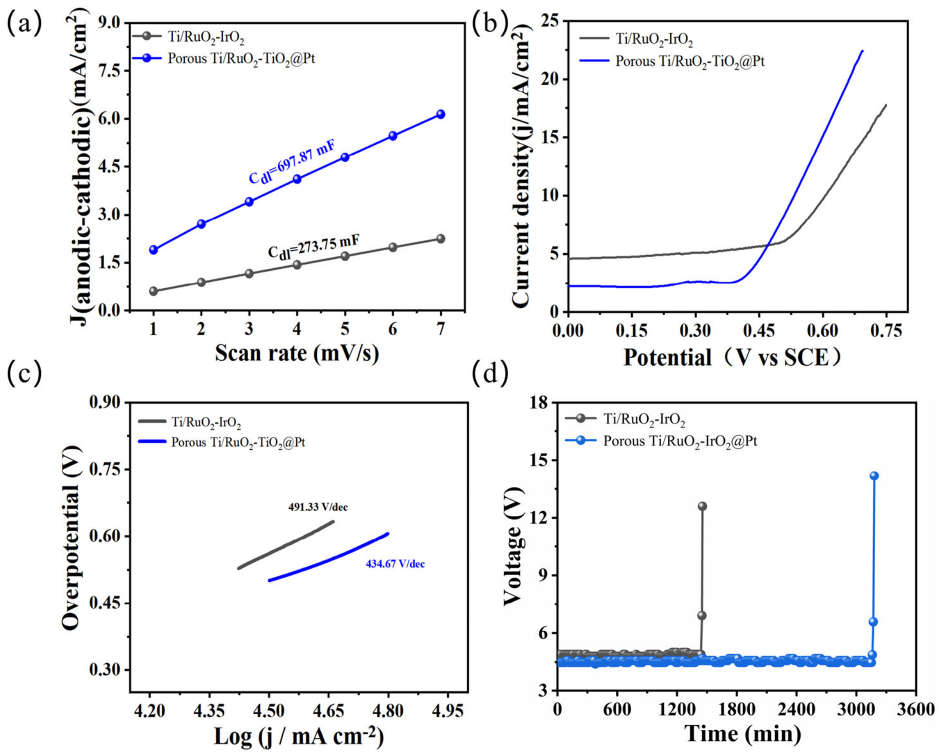


**Figure S5.** The electric double-layer capacitance diagram (a), LSV curves (b), Tafel curves (c) of porous Ti/$RuO_2$-$TiO_2$@Pt and Ti/$RuO_2$-$IrO_2$ electrodes. (d) Accelerated life test of the prepared electrodes at 2 A/$cm^2$ in 1 mol/L $H_2SO_4$ at 40 °C.

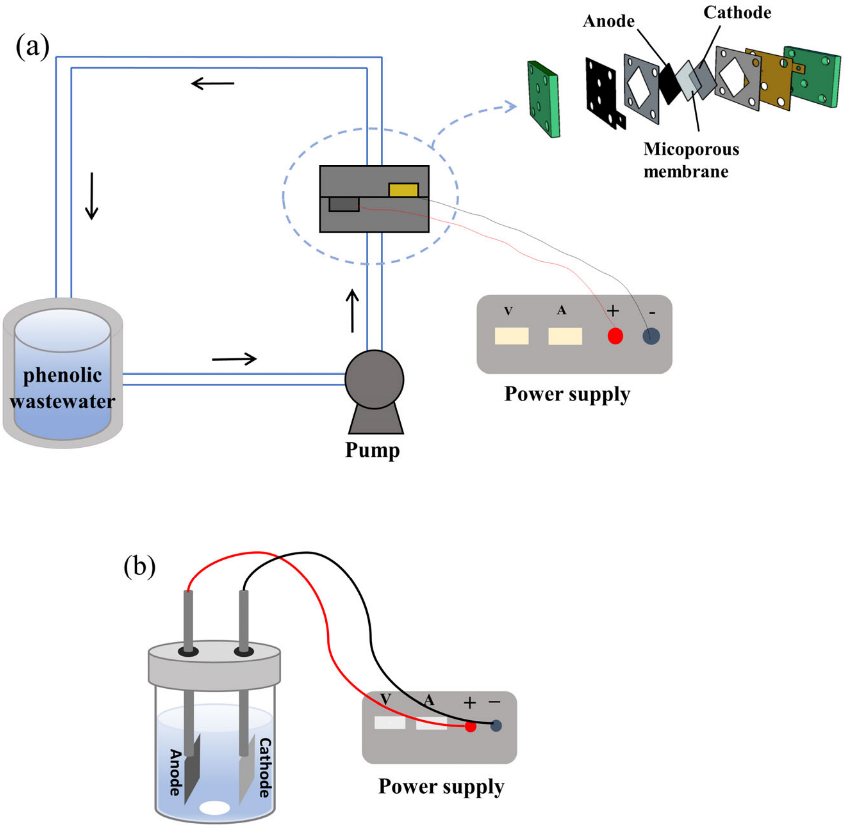


**Figure S6.** Scheme of the electrochemical systems. (a) zero-gap flow-through reactor; (b) conventional electrolyzer. Anode: 3D porous Ti/$RuO_2$-$IrO_2$@Pt and cathode: porous titanium plate.
